# (*Z*)-4-Hexyl-1-(5-nitro-2-oxo-2,3-dihydro-1*H*-indol-3-yl­idene)thio­semicarbazide

**DOI:** 10.1107/S1600536809040276

**Published:** 2009-10-10

**Authors:** Humayun Pervez, Muhammad Yaqub, Nazia Manzoor, M. Nawaz Tahir, M. Saeed Iqbal

**Affiliations:** a Department of Chemistry, Bahauddin Zakariya University, Multan 60800, Pakistan; b Department of Physics, University of Sargodha, Sargodha, Pakistan; c Department of Chemistry, Government College University, Lahore, Pakistan

## Abstract

In the title compound, C_15_H_19_N_5_O_3_S, intra­molecular N—H⋯O, N—H⋯N and C—H⋯S inter­actions occur and the three terminal C atoms of the hexyl group are disordered over two sites with an occupancy ratio of 0.664 (12):0.336 (12).  In the crystal, inversion dimers linked by pairs of N—H⋯O hydrogen bonds occur and C—H⋯O bonds link the dimers into chains. A short C=O⋯π contact is also present.

## Related literature

For the syntheses and structures of isatin and isatin-derived thio­semicarbazones with biological and medicinal properties, see: Beauchard *et al.* (2006 ▶); Hyatt *et al.* (2007 ▶); Quenelle *et al.* (2006 ▶); Karali *et al.* (2007 ▶). For a related crystal structure, see: Bain *et al.* (1997 ▶). For the syntheses of potent urease inhibitors based on *N*(4)-aryl­substituted isatin-3-thio­semicarbazones, see: Pervez *et al.* (2008 ▶, 2009 ▶). For the graph set analysis of hydrogen-bond patterns in crystal structures, see: Bernstein *et al.* (1995 ▶).
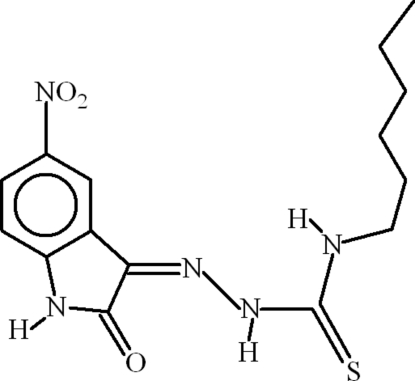


## Experimental

### 

#### Crystal data

C_15_H_19_N_5_O_3_S
*M* *_r_* = 349.42
Monoclinic, 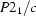

*a* = 11.9464 (6) Å
*b* = 4.8845 (3) Å
*c* = 29.9688 (17) Å
β = 101.131 (3)°
*V* = 1715.85 (17) Å^3^
*Z* = 4
Mo *K*α radiation
μ = 0.21 mm^−1^
*T* = 296 K
0.26 × 0.14 × 0.12 mm

#### Data collection

Bruker Kappa APEXII CCD diffractometer
Absorption correction: multi-scan (*SADABS*; Bruker, 2005 ▶) *T*_min_ = 0.963, *T*_max_ = 0.974
19438 measured reflections
4283 independent reflections
1964 reflections with *I* > 2σ(*I*)
*R*_int_ = 0.062

#### Refinement

*R*[*F* ^2^ > 2σ(*F* ^2^)] = 0.054
*wR*(*F* ^2^) = 0.143
*S* = 1.00
4283 reflections
247 parameters
6 restraints
H-atom parameters constrained
Δρ_max_ = 0.18 e Å^−3^
Δρ_min_ = −0.21 e Å^−3^

### 

Data collection: *APEX2* (Bruker, 2007 ▶); cell refinement: *SAINT* (Bruker, 2007 ▶); data reduction: *SAINT*; program(s) used to solve structure: *SHELXS97* (Sheldrick, 2008 ▶); program(s) used to refine structure: *SHELXL97* (Sheldrick, 2008 ▶); molecular graphics: *ORTEP-3 for Windows* (Farrugia, 1997 ▶) and *PLATON* (Spek, 2009 ▶); software used to prepare material for publication: *WinGX* (Farrugia, 1999 ▶) and *PLATON*.

## Supplementary Material

Crystal structure: contains datablocks global, I. DOI: 10.1107/S1600536809040276/si2206sup1.cif

Structure factors: contains datablocks I. DOI: 10.1107/S1600536809040276/si2206Isup2.hkl

Additional supplementary materials:  crystallographic information; 3D view; checkCIF report

## Figures and Tables

**Table 1:** Hydrogen-bond geometry (Å, °)

*D*—H⋯*A*	*D*—H	H⋯*A*	*D*⋯*A*	*D*—H⋯*A*
N2—H2*A*⋯O1^i^	0.86	2.08	2.871 (3)	153
N4—H4*A*⋯O1	0.86	2.02	2.721 (2)	138
N5—H5*A*⋯N3	0.86	2.32	2.688 (3)	106
C2—H2⋯O3^ii^	0.93	2.52	3.438 (3)	167
C10—H10*B*⋯O2^ii^	0.97	2.48	3.232 (4)	134
C7—O1⋯*Cg*1^iii^	1.23 (1)	3.25 (1)	3.896 (3)	113 (1)

Symmetry codes: (i) 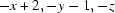; (ii) 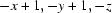; (iii) 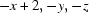. *Cg*1 is the centroid of the N2/C6/C1/C8/C7 ring.

**Fig. 1.**
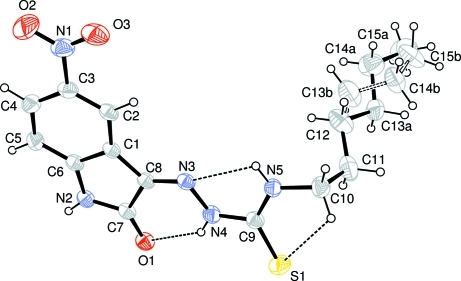
View of (I) with the atom numbering scheme. The thermal ellipsoids are drawn at the 30% probability level. H-atoms are shown by small circles of arbitrary radii. The single dotted lines represent the intramolecular H-bondings and the atoms of low occupancy factor are joined by double dotted lines.

**Fig. 2.**
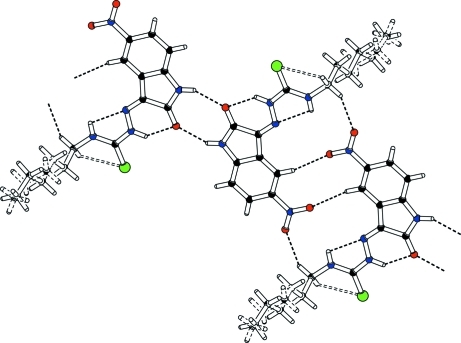
The partial packing (PLATON; Spek, 2009) which shows that the molecules form dimers which are joined in the form of polymeric chains.

## supplementary crystallographic information

### Comment

Isatin and its derivatives are known to possess a broad spectrum of pharmacological properties including antibacterial, anticonvulsant, antifungal, antineoplastic, antiviral, and enzymatic inhibition (Beauchard *et al.* 2006; Hyatt *et al.* 2007). Amongst these, isatins-derived thiosemicarbazones have gained a great deal of attention (Beauchard *et al.*, 2006; Quenelle *et al.*, 2006; Karali *et al.*, 2007). Very recently, a number of *N*(4)-arylsubstituted isatin-3-thiosemicarbazones have been synthesized and reported as potent urease (a nickel-dependent metallo-enzyme) inhibitors (Pervez *et al.*, 2008; Pervez *et al.*, 2009). In continuation to the development of potent and non- or less toxic urease inhibitors, we report herein the crystal structure and preparation of the title compound (I, Fig. 1).

The crystal structure of (II) Indole-2,3-dione 3-(*N*(4)-ethylthiosemicarbazone) (*N*^'^-2-(Thienylidene))benzhydrazide (Bain *et al.*, 1997) has been published. The title compound (I) differs from (II) due to hexyl moiety instead of ethyl moiety.

The molecules of the title compound consist of dimers owing to N–H···O type of intermolecular H-bondings forming *R*_2_^2^(8) ring motifs (Bernstein *et al.*, 1995). The molecules are interlinked in the form of polymeric chains due to C—H···O type of intermolecular H-bondings (Table 1, Fig. 2). There exist two S(5) and a S(6), *R*_2_^2^(10) and *R*_3_^3^(12) or *R*_2_^2^(13) ring motifs as well (Fig. 2). In the title compound the group (C1—C9/N1—N5/O—O3) of the isatin moiety along with nitro substitution is planar with maximum r.m.s. deviation of 0.0348 Å from the mean square plane and the sulphur atom S1 is at a distance of -0.3497 (16) Å from this mean square plane. The terminating three carbons of the hexyl group are disordered over two sites with occupancy ratio of 0.664 (12):0.336 (12). The C==O···π and N—O···π interactions (Table 1), may also be responsible for stabilizing of the molecules.

### Experimental

A solution of *N*^4^-hexylthiosemicarbazide (0.44 g, 2.5 mmol) in ethanol (10 ml) was added to a hot solution of 5-nitroisatin (0.48 g, 2.5 mmol) in 50% aqueous ethanol (30 ml) containing a few drops of glacial acetic acid. The reaction mixture was then heated under reflux for 2 h. The yellow crystalline solid formed during heating was collected by suction filtration. Thorough washing with hot aqueous ethanol gave the title compound (I) in pure form (0.72 g, 82%), m.p. 513 K. The single crystals of (I) were grown in ethanol-n-hexane (1:4) system by diffusion method at room temperature.

### Refinement

The H-atoms were positioned geometrically with N—H = 0.86, C—H = 0.93, 0.96 and 0.97 Å for aryl, methyl and methylene H atoms respectively and constrained to ride on their parent atoms, with *U*_iso_(H) = *xU*_eq_(C, N), where *x* = 1.5 for methyl and 1.2 for all other H atoms.

### Crystal data

**Table:**

C_15_H_19_N_5_O_3_S	*F*(000) = 736
*M**_r_* = 349.42	*D*_x_ = 1.353 Mg m^−^^3^
Monoclinic, *P*2_1_/*c*	Mo *K*α radiation, λ = 0.71073 Å
Hall symbol: -P 2ybc	Cell parameters from 4283 reflections
*a* = 11.9464 (6) Å	θ = 2.8–28.3°
*b* = 4.8845 (3) Å	µ = 0.21 mm^−^^1^
*c* = 29.9688 (17) Å	*T* = 296 K
β = 101.131 (3)°	Needle, yellow
*V* = 1715.85 (17) Å^3^	0.26 × 0.14 × 0.12 mm
*Z* = 4	

### Data collection

**Table:**

Bruker Kappa APEXII CCD diffractometer	4283 independent reflections
Radiation source: fine-focus sealed tube	1964 reflections with *I* > 2σ(*I*)
graphite	*R*_int_ = 0.062
Detector resolution: 7.40 pixels mm^-1^	θ_max_ = 28.3°, θ_min_ = 2.8°
ω scans	*h* = −15→15
Absorption correction: multi-scan (*SADABS*; Bruker, 2005)	*k* = −6→6
*T*_min_ = 0.963, *T*_max_ = 0.974	*l* = −39→39
19438 measured reflections	

### Refinement

**Table:**

Refinement on *F*^2^	Primary atom site location: structure-invariant direct methods
Least-squares matrix: full	Secondary atom site location: difference Fourier map
*R*[*F*^2^ > 2σ(*F*^2^)] = 0.054	Hydrogen site location: inferred from neighbouring sites
*wR*(*F*^2^) = 0.143	H-atom parameters constrained
*S* = 1.00	*w* = 1/[σ^2^(*F*_o_^2^) + (0.0588*P*)^2^] where *P* = (*F*_o_^2^ + 2*F*_c_^2^)/3
4283 reflections	(Δ/σ)_max_ = 0.001
247 parameters	Δρ_max_ = 0.18 e Å^−^^3^
6 restraints	Δρ_min_ = −0.20 e Å^−^^3^

### Special details

**Table:**

Geometry. Bond distances, angles *etc*. have been calculated using the rounded fractional coordinates. All su's are estimated from the variances of the (full) variance-covariance matrix. The cell e.s.d.'s are taken into account in the estimation of distances, angles and torsion angles
Refinement. Refinement of *F*^2^ against ALL reflections. The weighted *R*-factor *wR* and goodness of fit *S* are based on *F*^2^, conventional *R*-factors *R* are based on *F*, with *F* set to zero for negative *F*^2^. The threshold expression of *F*^2^ > σ(*F*^2^) is used only for calculating *R*-factors(gt) *etc*. and is not relevant to the choice of reflections for refinement. *R*-factors based on *F*^2^ are statistically about twice as large as those based on *F*, and *R*- factors based on ALL data will be even larger.

### Fractional atomic coordinates and isotropic or equivalent isotropic displacement parameters (Å^2^)

**Table:**

	*x*	*y*	*z*	*U*_iso_*/*U*_eq_	Occ. (<1)
S1	1.08831 (6)	0.36613 (17)	0.14896 (3)	0.0774 (3)	
O1	0.99569 (14)	−0.2388 (3)	0.03674 (5)	0.0529 (6)	
O2	0.3667 (2)	0.1096 (6)	−0.11002 (9)	0.1287 (13)	
O3	0.41037 (17)	0.3726 (5)	−0.05287 (8)	0.0877 (9)	
N1	0.4324 (2)	0.1883 (6)	−0.07641 (10)	0.0709 (10)	
N2	0.85164 (16)	−0.3241 (4)	−0.02531 (7)	0.0490 (7)	
N3	0.83033 (16)	0.1924 (4)	0.05490 (7)	0.0458 (7)	
N4	0.93056 (16)	0.1876 (4)	0.08428 (7)	0.0510 (8)	
N5	0.87198 (16)	0.5295 (4)	0.12710 (6)	0.0503 (7)	
C1	0.71964 (19)	−0.0082 (4)	−0.01440 (8)	0.0433 (8)	
C2	0.6158 (2)	0.1275 (5)	−0.02523 (8)	0.0484 (8)	
C3	0.5423 (2)	0.0476 (5)	−0.06399 (9)	0.0542 (9)	
C4	0.5668 (2)	−0.1580 (5)	−0.09235 (9)	0.0605 (10)	
C5	0.6693 (2)	−0.2938 (5)	−0.08188 (9)	0.0568 (10)	
C6	0.7441 (2)	−0.2180 (4)	−0.04288 (8)	0.0459 (8)	
C7	0.9006 (2)	−0.1945 (4)	0.01354 (8)	0.0448 (8)	
C8	0.81712 (19)	0.0150 (4)	0.02204 (8)	0.0427 (8)	
C9	0.9565 (2)	0.3694 (5)	0.11984 (9)	0.0496 (8)	
C10	0.8834 (2)	0.7192 (5)	0.16504 (9)	0.0623 (10)	
C11	0.8653 (3)	0.5848 (7)	0.20820 (10)	0.0824 (14)	
C12	0.7498 (3)	0.4789 (8)	0.20746 (12)	0.0955 (17)	
C13A	0.7475 (5)	0.3834 (17)	0.2565 (2)	0.077 (3)	0.664 (12)
C14A	0.6340 (6)	0.279 (2)	0.2605 (3)	0.102 (3)	0.664 (12)
C15A	0.6209 (16)	0.201 (4)	0.3075 (5)	0.127 (6)	0.664 (12)
C15B	0.635 (3)	0.256 (6)	0.3121 (11)	0.109 (10)	0.336 (12)
C13B	0.6968 (19)	0.273 (3)	0.2355 (6)	0.112 (7)	0.336 (12)
C14B	0.6793 (19)	0.418 (3)	0.2762 (4)	0.094 (6)	0.336 (12)
H2	0.59683	0.26707	−0.00692	0.0581*	
H2A	0.88306	−0.45536	−0.03755	0.0587*	
H4	0.51425	−0.20417	−0.11838	0.0726*	
H4A	0.98036	0.06652	0.08074	0.0612*	
H10B	0.82826	0.86578	0.15726	0.0747*	
H11A	0.88335	0.71653	0.23276	0.0988*	
H11B	0.91888	0.43442	0.21509	0.0988*	
H12A	0.69345	0.62106	0.19818	0.1147*	
H12B	0.73346	0.32686	0.18638	0.1147*	
H13A	0.76759	0.53567	0.27726	0.0930*	0.664 (12)
H13B	0.80392	0.24041	0.26509	0.0930*	0.664 (12)
H14A	0.57765	0.41744	0.24905	0.1224*	0.664 (12)
H14B	0.61727	0.11930	0.24100	0.1224*	0.664 (12)
H15A	0.61520	0.36407	0.32492	0.1907*	0.664 (12)
H15B	0.55311	0.09342	0.30598	0.1907*	0.664 (12)
H15C	0.68605	0.09686	0.32190	0.1907*	0.664 (12)
H5	0.68764	−0.43231	−0.10052	0.0681*	
H5A	0.80755	0.52050	0.10849	0.0603*	
H10A	0.95894	0.79989	0.17013	0.0747*	
H13C	0.74739	0.11816	0.24371	0.1345*	0.336 (12)
H13D	0.62469	0.20663	0.21835	0.1345*	0.336 (12)
H14C	0.75141	0.49973	0.29030	0.1130*	0.336 (12)
H14D	0.62625	0.56648	0.26657	0.1130*	0.336 (12)
H15D	0.67630	0.30834	0.34164	0.1633*	0.336 (12)
H15E	0.55557	0.29260	0.30999	0.1633*	0.336 (12)
H15F	0.64630	0.06410	0.30749	0.1633*	0.336 (12)

### Atomic displacement parameters (Å^2^)

**Table:**

	*U*^11^	*U*^22^	*U*^33^	*U*^12^	*U*^13^	*U*^23^
S1	0.0496 (5)	0.0958 (6)	0.0813 (6)	0.0104 (4)	−0.0009 (4)	−0.0106 (4)
O1	0.0500 (11)	0.0514 (10)	0.0578 (11)	0.0164 (8)	0.0117 (9)	0.0052 (8)
O2	0.0754 (17)	0.194 (3)	0.1011 (19)	0.0471 (16)	−0.0218 (15)	−0.0188 (19)
O3	0.0657 (14)	0.0890 (15)	0.1102 (18)	0.0332 (11)	0.0215 (13)	0.0064 (13)
N1	0.0488 (15)	0.093 (2)	0.0701 (17)	0.0148 (14)	0.0096 (14)	0.0203 (15)
N2	0.0509 (13)	0.0428 (11)	0.0559 (13)	0.0130 (9)	0.0171 (10)	−0.0032 (10)
N3	0.0427 (12)	0.0470 (12)	0.0495 (12)	0.0089 (9)	0.0136 (10)	0.0063 (10)
N4	0.0472 (13)	0.0520 (13)	0.0538 (13)	0.0136 (9)	0.0097 (11)	−0.0028 (10)
N5	0.0474 (12)	0.0554 (12)	0.0465 (12)	0.0073 (10)	0.0054 (10)	−0.0050 (10)
C1	0.0450 (15)	0.0396 (12)	0.0485 (14)	0.0062 (11)	0.0170 (12)	0.0059 (11)
C2	0.0480 (15)	0.0466 (13)	0.0547 (16)	0.0125 (11)	0.0201 (13)	0.0062 (12)
C3	0.0433 (15)	0.0621 (17)	0.0588 (17)	0.0075 (12)	0.0136 (14)	0.0118 (14)
C4	0.0526 (17)	0.0727 (19)	0.0545 (17)	−0.0010 (14)	0.0062 (14)	0.0036 (14)
C5	0.0601 (18)	0.0592 (16)	0.0523 (16)	0.0038 (14)	0.0142 (14)	−0.0077 (13)
C6	0.0468 (15)	0.0418 (13)	0.0524 (15)	0.0066 (11)	0.0177 (12)	0.0059 (12)
C7	0.0469 (15)	0.0405 (13)	0.0498 (15)	0.0092 (11)	0.0167 (13)	0.0077 (12)
C8	0.0432 (14)	0.0400 (13)	0.0475 (14)	0.0092 (11)	0.0153 (12)	0.0049 (11)
C9	0.0478 (15)	0.0524 (14)	0.0497 (15)	0.0064 (12)	0.0122 (12)	0.0052 (12)
C10	0.0660 (18)	0.0582 (16)	0.0613 (18)	0.0066 (13)	0.0092 (14)	−0.0105 (14)
C11	0.100 (3)	0.091 (2)	0.057 (2)	0.0197 (19)	0.0171 (18)	−0.0106 (17)
C12	0.108 (3)	0.099 (3)	0.093 (3)	0.007 (2)	0.053 (2)	0.009 (2)
C13A	0.079 (4)	0.104 (5)	0.049 (4)	−0.012 (3)	0.012 (3)	−0.008 (3)
C14A	0.089 (5)	0.130 (7)	0.090 (6)	−0.017 (4)	0.023 (4)	0.023 (5)
C15A	0.113 (9)	0.188 (14)	0.078 (7)	−0.020 (7)	0.012 (6)	0.033 (8)
C15B	0.112 (18)	0.107 (12)	0.13 (2)	0.020 (12)	0.079 (16)	−0.002 (12)
C13B	0.142 (15)	0.096 (9)	0.115 (13)	−0.021 (9)	0.068 (12)	−0.024 (9)
C14B	0.123 (14)	0.089 (10)	0.081 (9)	−0.006 (8)	0.047 (10)	−0.012 (7)

### Geometric parameters (Å, °)

**Table:**

S1—C9	1.647 (3)	C13A—C14A	1.475 (10)
O1—C7	1.231 (3)	C13B—C14B	1.46 (2)
O2—N1	1.214 (4)	C14A—C15A	1.496 (18)
O3—N1	1.204 (4)	C14B—C15B	1.51 (4)
N1—C3	1.465 (4)	C2—H2	0.9300
N2—C6	1.391 (3)	C4—H4	0.9300
N2—C7	1.355 (3)	C5—H5	0.9300
N3—N4	1.343 (3)	C10—H10A	0.9700
N3—C8	1.298 (3)	C10—H10B	0.9700
N4—C9	1.376 (3)	C11—H11A	0.9700
N5—C9	1.328 (3)	C11—H11B	0.9700
N5—C10	1.453 (3)	C12—H12A	0.9700
N2—H2A	0.8600	C12—H12B	0.9700
N4—H4A	0.8600	C13A—H13A	0.9700
N5—H5A	0.8600	C13A—H13B	0.9700
C1—C2	1.389 (3)	C13B—H13D	0.9700
C1—C8	1.439 (3)	C13B—H13C	0.9700
C1—C6	1.400 (3)	C14A—H14B	0.9700
C2—C3	1.371 (4)	C14A—H14A	0.9700
C3—C4	1.383 (4)	C14B—H14C	0.9700
C4—C5	1.375 (3)	C14B—H14D	0.9700
C5—C6	1.378 (3)	C15A—H15B	0.9600
C7—C8	1.485 (3)	C15A—H15C	0.9600
C10—C11	1.503 (4)	C15A—H15A	0.9600
C11—C12	1.470 (5)	C15B—H15D	0.9600
C12—C13B	1.524 (18)	C15B—H15E	0.9600
C12—C13A	1.547 (7)	C15B—H15F	0.9600
			
S1···C11	3.636 (4)	C15A···H5^xii^	3.0200
S1···H10A	2.7700	C15B···H5^xii^	3.0200
S1···H11B	3.1100	H2···O3^vi^	2.5200
S1···H13A^i^	3.0000	H2···O3	2.4400
S1···H15C^ii^	2.9000	H2A···O1^v^	2.0800
S1···H14C^i^	2.9700	H2A···C7^v^	3.0700
O1···N3	3.008 (3)	H4···O2	2.3900
O1···N4	2.721 (2)	H4A···O1	2.0200
O1···C9^iii^	3.246 (3)	H4A···C7	2.4200
O1···C7^iv^	3.001 (3)	H5···H15C^xiii^	2.4600
O1···O1^iv^	3.223 (2)	H5···C15B^xiii^	3.0200
O1···N2^v^	2.871 (3)	H5···C15A^xiii^	3.0200
O1···C8^iv^	3.289 (3)	H5···H15D^xiii^	2.5100
O2···C10^vi^	3.232 (4)	H5A···N3	2.3200
O3···C4^vii^	3.317 (3)	H5A···O2^vi^	2.7600
O3···C1^viii^	3.295 (3)	H5A···O3^vi^	2.8600
O1···H4A	2.0200	H10A···S1	2.7700
O1···H2A^v^	2.0800	H10B···O2^vi^	2.4800
O2···H10B^vi^	2.4800	H10B···H12A	2.5100
O2···H12A^vi^	2.9100	H11A···H13A	2.2800
O2···H5A^vi^	2.7600	H11B···S1	3.1100
O2···H4	2.3900	H11B···H13B	2.4100
O3···H2	2.4400	H11B···C9	2.9900
O3···H2^vi^	2.5200	H12A···H10B	2.5100
O3···H5A^vi^	2.8600	H12A···H14D	2.3600
N2···O1^v^	2.871 (3)	H12A···O2^vi^	2.9100
N3···O1	3.008 (3)	H12A···H14A	2.4600
N3···N5	2.688 (3)	H12B···N5	2.8300
N3···C7^vii^	3.408 (3)	H12B···H14B	2.5500
N4···O1	2.721 (2)	H13A···H11A	2.2800
N5···N3	2.688 (3)	H13A···S1^ii^	3.0000
N3···H5A	2.3200	H13B···H15C	2.5100
N5···H12B	2.8300	H13B···H11B	2.4100
C1···O3^viii^	3.295 (3)	H13C···H15F	2.4600
C2···C5^vii^	3.421 (3)	H14A···C14A^xi^	3.0500
C4···O3^iii^	3.317 (3)	H14A···C15A^xi^	2.9800
C5···C2^iii^	3.421 (3)	H14A···H15B^xi^	2.2100
C7···N3^iii^	3.408 (3)	H14A···H14B^xi^	2.6000
C7···C7^iv^	3.264 (3)	H14A···H12A	2.4600
C7···O1^iv^	3.001 (3)	H14B···H12B	2.5500
C8···O1^iv^	3.289 (3)	H14B···H14A^x^	2.6000
C9···O1^vii^	3.246 (3)	H14C···C11	3.0600
C10···O2^vi^	3.232 (4)	H14C···S1^ii^	2.9700
C11···S1	3.636 (4)	H14D···H12A	2.3600
C4···H15D^ix^	3.0900	H15A···C4^xiv^	3.0200
C4···H15A^ix^	3.0200	H15B···C14A^x^	3.1000
C7···H2A^v^	3.0700	H15B···H14A^x^	2.2100
C7···H4A	2.4200	H15C···S1^i^	2.9000
C9···H11B	2.9900	H15C···H5^xii^	2.4600
C11···H14C	3.0600	H15C···H13B	2.5100
C14A···H14A^x^	3.0500	H15D···C4^xiv^	3.0900
C14A···H15B^xi^	3.1000	H15D···H5^xii^	2.5100
C15A···H14A^x^	2.9800	H15F···H13C	2.4600
			
O2—N1—O3	122.9 (3)	N5—C10—H10B	109.00
O2—N1—C3	117.8 (3)	C11—C10—H10A	109.00
O3—N1—C3	119.3 (3)	C11—C10—H10B	109.00
C6—N2—C7	111.42 (19)	H10A—C10—H10B	108.00
N4—N3—C8	116.6 (2)	C10—C11—H11A	108.00
N3—N4—C9	122.4 (2)	C10—C11—H11B	108.00
C9—N5—C10	123.0 (2)	C12—C11—H11A	108.00
C7—N2—H2A	124.00	C12—C11—H11B	108.00
C6—N2—H2A	124.00	H11A—C11—H11B	107.00
N3—N4—H4A	119.00	C11—C12—H12A	110.00
C9—N4—H4A	119.00	C11—C12—H12B	110.00
C10—N5—H5A	119.00	C13A—C12—H12A	110.00
C9—N5—H5A	119.00	C13A—C12—H12B	110.00
C2—C1—C6	119.3 (2)	H12A—C12—H12B	109.00
C6—C1—C8	106.66 (19)	C13B—C12—H12A	107.00
C2—C1—C8	134.1 (2)	C13B—C12—H12B	79.00
C1—C2—C3	117.4 (2)	C12—C13A—H13A	109.00
C2—C3—C4	123.4 (2)	C12—C13A—H13B	109.00
N1—C3—C4	118.0 (2)	C14A—C13A—H13A	109.00
N1—C3—C2	118.5 (2)	C14A—C13A—H13B	109.00
C3—C4—C5	119.7 (2)	H13A—C13A—H13B	108.00
C4—C5—C6	117.9 (2)	H13C—C13B—H13D	109.00
N2—C6—C1	109.2 (2)	C12—C13B—H13C	110.00
N2—C6—C5	128.4 (2)	C12—C13B—H13D	110.00
C1—C6—C5	122.4 (2)	C14B—C13B—H13C	110.00
O1—C7—C8	127.0 (2)	C14B—C13B—H13D	110.00
O1—C7—N2	127.0 (2)	C15A—C14A—H14A	109.00
N2—C7—C8	106.1 (2)	C15A—C14A—H14B	108.00
N3—C8—C1	126.2 (2)	C13A—C14A—H14B	108.00
N3—C8—C7	127.1 (2)	C13A—C14A—H14A	108.00
C1—C8—C7	106.71 (19)	H14A—C14A—H14B	107.00
N4—C9—N5	116.3 (2)	C13B—C14B—H14C	108.00
S1—C9—N5	126.8 (2)	C13B—C14B—H14D	108.00
S1—C9—N4	116.98 (18)	H14C—C14B—H14D	107.00
N5—C10—C11	112.8 (2)	C15B—C14B—H14D	108.00
C10—C11—C12	115.6 (3)	C15B—C14B—H14C	108.00
C11—C12—C13B	135.4 (8)	C14A—C15A—H15C	110.00
C11—C12—C13A	106.3 (3)	C14A—C15A—H15B	110.00
C12—C13A—C14A	111.5 (5)	H15B—C15A—H15C	110.00
C12—C13B—C14B	106.4 (11)	H15A—C15A—H15B	109.00
C13A—C14A—C15A	115.4 (9)	H15A—C15A—H15C	109.00
C13B—C14B—C15B	117.9 (17)	C14A—C15A—H15A	109.00
C1—C2—H2	121.00	C14B—C15B—H15D	109.00
C3—C2—H2	121.00	C14B—C15B—H15E	110.00
C3—C4—H4	120.00	C14B—C15B—H15F	109.00
C5—C4—H4	120.00	H15D—C15B—H15E	110.00
C4—C5—H5	121.00	H15D—C15B—H15F	109.00
C6—C5—H5	121.00	H15E—C15B—H15F	110.00
N5—C10—H10A	109.00		
			
O2—N1—C3—C2	−177.7 (3)	C8—C1—C6—C5	−179.2 (2)
O2—N1—C3—C4	2.9 (4)	C2—C1—C8—N3	−2.5 (4)
O3—N1—C3—C2	1.3 (4)	C2—C1—C8—C7	179.8 (2)
O3—N1—C3—C4	−178.1 (3)	C6—C1—C8—N3	177.5 (2)
C7—N2—C6—C1	−0.2 (3)	C6—C1—C8—C7	−0.2 (2)
C7—N2—C6—C5	179.2 (2)	C1—C2—C3—N1	−179.6 (2)
C6—N2—C7—O1	−179.0 (2)	C1—C2—C3—C4	−0.2 (4)
C6—N2—C7—C8	0.1 (3)	N1—C3—C4—C5	179.7 (2)
C8—N3—N4—C9	178.2 (2)	C2—C3—C4—C5	0.3 (4)
N4—N3—C8—C1	−176.8 (2)	C3—C4—C5—C6	0.2 (4)
N4—N3—C8—C7	0.5 (3)	C4—C5—C6—N2	179.9 (2)
N3—N4—C9—S1	−172.11 (18)	C4—C5—C6—C1	−0.7 (4)
N3—N4—C9—N5	8.0 (3)	O1—C7—C8—N3	1.5 (4)
C10—N5—C9—S1	−4.1 (4)	O1—C7—C8—C1	179.2 (2)
C10—N5—C9—N4	175.8 (2)	N2—C7—C8—N3	−177.6 (2)
C9—N5—C10—C11	−83.5 (3)	N2—C7—C8—C1	0.1 (2)
C6—C1—C2—C3	−0.3 (3)	N5—C10—C11—C12	−65.1 (3)
C8—C1—C2—C3	179.7 (2)	C10—C11—C12—C13A	−173.3 (4)
C2—C1—C6—N2	−179.7 (2)	C11—C12—C13A—C14A	178.4 (6)
C2—C1—C6—C5	0.8 (3)	C12—C13A—C14A—C15A	−175.7 (10)
C8—C1—C6—N2	0.3 (2)		

Symmetry codes: (i) −*x*+2, *y*−1/2, −*z*+1/2; (ii) −*x*+2, *y*+1/2, −*z*+1/2; (iii) *x*, *y*−1, *z*; (iv) −*x*+2, −*y*, −*z*; (v) −*x*+2, −*y*−1, −*z*; (vi) −*x*+1, −*y*+1, −*z*; (vii) *x*, *y*+1, *z*; (viii) −*x*+1, −*y*, −*z*; (ix) *x*, −*y*+1/2, *z*−1/2; (x) −*x*+1, *y*−1/2, −*z*+1/2; (xi) −*x*+1, *y*+1/2, −*z*+1/2; (xii) *x*, −*y*−1/2, *z*+1/2; (xiii) *x*, −*y*−1/2, *z*−1/2; (xiv) *x*, −*y*+1/2, *z*+1/2.

### Hydrogen-bond geometry (Å, °)

**Table:**

*D*—H···*A*	*D*—H	H···*A*	*D*···*A*	*D*—H···*A*
N2—H2A···O1^v^	0.8600	2.0800	2.871 (3)	153.00
N4—H4A···O1	0.8600	2.0200	2.721 (2)	138.00
N5—H5A···N3	0.8600	2.3200	2.688 (3)	106.00
C2—H2···O3^vi^	0.9300	2.5200	3.438 (3)	167.00
C10—H10A···S1	0.9700	2.7700	3.107 (3)	101.00
C10—H10B···O2^vi^	0.9700	2.4800	3.232 (4)	134.00
C7—O1···Cg1^iv^	1.231 (3)	3.245 (2)	3.896 (3)	113.13 (13)
N1—O3···Cg1^viii^	1.204 (4)	3.646 (2)	4.201 (3)	109.29 (18)
N1—O3···Cg2^viii^	1.204 (4)	3.652 (3)	4.192 (3)	108.50 (19)

Symmetry codes: (v) −*x*+2, −*y*−1, −*z*; (vi) −*x*+1, −*y*+1, −*z*; (iv) −*x*+2, −*y*, −*z*; (viii) −*x*+1, −*y*, −*z*.
